# A critical scoping analysis of digital media literacy research in selected Arab and international journals (2016–2025)

**DOI:** 10.3389/frma.2026.1746718

**Published:** 2026-03-23

**Authors:** Mohammad Ali Alquaary, Osama Abdelrheem Ali, Hossam Fayez

**Affiliations:** 1 Journalism and New Media Department, College of Media and Communication, Imam Mohammad Ibn Saud Islamic University (IMSIU), Riyadh, Saudi Arabia; 2 Media Department, Faculty of Specific Education, Mansoura University, Mansoura, Egypt; 3 Media Department, Faculty of Specific Education, Minia University, Minya, Egypt

**Keywords:** Arab and international journals, critical analysis, digital media literacy, information literacy, qualitative approach

## Abstract

**Introduction:**

Digital media literacy has emerged as a critical field of inquiry in response to the rapid expansion of digital communication technologies and social media platforms. Despite the growing importance of this field, scholarly production remains uneven across different academic contexts. This study aims to provide an in-depth analytical examination of contemporary trends in digital media literacy research by analyzing publications in selected Arab and international peer-reviewed journals.

**Methods:**

The study adopts a secondary data analysis approach to systematically examine published research in four peer-reviewed journals—two Arab and two international—over a 10-year period from 2016 to mid-2025. This method, widely recognized in qualitative research, was employed to analyze the theoretical orientations, methodological approaches, and thematic patterns of the selected studies, as well as to identify existing research gaps within the field.

**Results:**

The findings reveal a clear imbalance in scholarly contributions, with international journals accounting for 66.5% of the analyzed studies compared to 33.5% in Arab journals. The majority of the reviewed research focused on educational and media-related topics (70%), while other themes received comparatively limited attention. Furthermore, the analysis shows that approximately 73% of the studies lacked a clearly articulated theoretical framework, which limits the development of a solid epistemological and philosophical foundation for the field. The results also indicate that research in digital media literacy remains predominantly descriptive, with limited methodological diversity.

**Discussion:**

The findings highlight the need to strengthen theoretical and methodological rigor in digital media literacy research, particularly within the Arab academic context. Developing more diversified theoretical frameworks that incorporate broader social systems is essential for advancing the field. The study also underscores the importance of employing more varied research methodologies, including qualitative and experimental approaches.

## 1 Introduction

In recent decades, the media and communication landscape has undergone profound transformations with the rise of digital platforms and the growing migration of audiences, particularly younger generations, toward interactive digital environments. These shifts have called for a redefinition of media literacy concepts and frameworks to align with contemporary contexts of media consumption and production. Media literacy is no longer limited to understanding and analyzing media messages; it has evolved to encompass digital participation skills, information verification, critical thinking, and digital citizenship (d'Haenens and Ioris, 2025). In this context, the concept of *digital media literacy* has emerged as a multidisciplinary field at the intersection of media, education, cultural studies, and technology. This conceptual expansion has been reflected in a significant increase in scholarly output addressing issues such as media manipulation, the impact of algorithms, the use of artificial intelligence, and patterns of digital interaction among children and young people (Prastyanti et al., 2025).

Despite this body of research, the field of digital media literacy still suffers from clear fragmentation in its thematic approaches, disparities in the theoretical frameworks employed, and a notable dominance of descriptive research at the expense of interpretive and critical approaches (Eyal and Te'eni-Harari, 2024). Previous reviews also indicate the limited number of studies that offer comparative analytical readings across different publication contexts (Dridi, 2023; Polanco-Levicán and Salvo-Garrido, 2022; Wuyckens et al., 2022), particularly between Arabic literature and its counterparts published in international journals.

In light of this, the need arises for analytical studies that examine the structure of scholarly output in the field of digital media literacy, focusing on the topics addressed, methodological designs, and the degree of application of theoretical frameworks, without claiming to offer a comprehensive methodological review or meta-analysis. This approach gains particular importance when applied to selected scholarly journals representing diverse Arab and international publication contexts.

Based on this premise, the current study adopts a critical, exploratory, analytical approach, aiming to provide a structured analytical reading of published research on digital media literacy, focusing on prevailing research trends, knowledge gaps, and the limitations of theoretical and methodological development in this field.

This study does not aim to provide a comprehensive survey or a complete representation of the field of digital media literacy studies globally. Rather, it seeks to conduct an in-depth critical analysis of published scholarly output within specific and intentional publishing contexts. The study's scope has been limited to academic journals rather than broad databases to examine research patterns and trends within specific editorial and academic environments. Although the study engages in a critical reading of published literature, its focus is on describing and analyzing research patterns and trends within selected journals, rather than reconstructing study results or testing causal relationships across studies. This distinguishes it from a systematic review and meta-analytical.

## 2 Study purpose and research questions

The study aims to:

- Map and analyze current research trends in digital media literacy studies published in Arab and international journals included in the sample.
- Analyze the prevailing methodological characteristics in this research, including research designs and data collection tools.
- Examine the extent to which theoretical frameworks and models are employed in digital media literacy studies.
- Conduct an analytical comparison between Arab and international publishing contexts in terms of research priorities and methodological approaches.

Based on these objectives, this study seeks to answer the following research questions:

1. What are the most prominent topics addressed in digital media literacy research in the journals under study?
2. What are the most common methodological designs in this research?
3. To what extent do digital media literacy studies rely on clear theoretical frameworks or models?
4. What are the similarities and differences between studies published in Arab journals and their international counterparts?

## 3 Literature review

The literature review in this study does not aim to list or chronologically review previous studies, but rather to provide a critical synthesis that reveals prevailing theoretical and methodological trends, conceptual tensions, and knowledge gaps in digital media literacy research. Accordingly, the literature is organized according to analytical dimensions that reflect the nature of the research questions, the level of theoretical grounding, and the patterns of methodological design, rather than simply presenting a descriptive overview.

### 3.1 Review of digital media literacy research: trends, orientations, and gaps

Rather than providing a chronological summary of prior studies, this section synthesizes key research trends in digital media literacy by identifying dominant thematic orientations, methodological patterns, and conceptual limitations. The reviewed studies are grouped analytically to highlight areas of convergence, divergence, and undertheorization, thereby situating the current study within existing review scholarship and clarifying the specific contribution it seeks to make.

Previous research analyzing the literature on digital media literacy has been diverse due to the breadth of the concept itself (Abdelhay et al., 2025) and the variety of media examined across educational, technological, and social contexts. Among studies exploring the concept of media literacy, Hanafi (2023) examined the evolution of digital media literacy in Arab and international research. The study found that much of the research emphasized digital literacy and its skills, particularly during the COVID-19 pandemic, which highlighted the need to integrate digital media literacy into curricula and lectures amid the widespread adoption of remote learning. The findings also indicated that digital literacy extends beyond the humanities and media fields, intersecting with other disciplines such as computer science and engineering. Similarly, Schofield et al. (2023) reviewed studies on media and information literacy (MIL) and found that many adopted a broad understanding of the concept, often based on initiatives by international organizations such as UNESCO. In the same vein, ghander (2023) conducted a descriptive analysis of definitions and core competencies of social media literacy through a systematic literature review across databases such as Scopus, Web of Science, and PubMed. The results indicated that social media literacy builds on traditional media literacy while incorporating the features and influences of digital platforms. It emphasizes the development of cognitive, social-emotional, and technical competencies within their social contexts. The study also noted that most research on social media literacy is concentrated in European countries.

In light of the growing interest in research trends on media literacy, Mekawey et al. (2021) analyzed the various research directions and their evolution in this field. The study revealed a global expansion of media literacy research and showed that the definition of key concepts remains the dominant research trend, reflecting the field's dynamic nature and its adaptation to the constantly changing media environment. The study also noted that international research has paid significant attention to the role of civil society and non-governmental organizations in promoting media literacy.

Wuyckens et al. (2022) conducted a comprehensive systematic review of the literature addressing information literacy, media literacy, and digital literacy, examining how these interrelated concepts are defined and discussed. Their findings revealed that literacy-related concepts draw on multiple disciplines, which gives them epistemological depth. Literature has expanded the foundational dimensions of literacy traditionally limited to reading and writing to include all forms of modern media communication. Consequently, information literacy, media literacy, and digital literacy coexist within an evolving conceptual environment alongside critical media literacy. Among studies employing a methodological review approach, Ahmad Tajuddin et al. (2024) identified the most prominent indicators in media literacy research. The results showed that media literacy is a continuous, cumulative, and lifelong process encompassing all age groups. It involves conscious access to media messages, critical analysis, and the ability to create and share content responsibly. The study also pointed to a clear deficiency in implementing media literacy initiatives across Arab societies.

Demir and Akar (2024) conducted a systematic review of literature on *parental engagement in digital education*, exploring parents' strategies and competencies as reflected in prior studies. The review found that parents in digital learning contexts could be classified into groups that either fear digital tools or experience knowledge gaps, with many expressing a need for support and guidance in digital education. Haywood and Sembiante (2023) also reviewed the literature on teaching media literacy to parents and emphasized the importance of assessing parental needs and fostering openness to media literacy education. They concluded that integrating media literacy instruction with parental mediation training promotes healthy development, positive communication, and social interaction among children, while also preventing behavioral issues and enhancing family dynamics. Finally, Zhang et al. (2020) reviewed literature on integrating media literacy into European curricula, focusing on the main objectives of media literacy education. Their analysis identified three core goals: understanding media, producing media content, and developing critical awareness of media systems.

#### 3.1.1 Knowledge gap and analytical implications

The reviewed studies reveal that the conceptual diversity across the fields of media, information, and digital literacy presents a major challenge to cumulative knowledge and the development of unified theoretical frameworks. This multiplicity highlights the lack of consensus among researchers on precise definitions and the specific competencies to be included. The fluidity of these concepts often results in ambiguity when setting educational and social objectives. Consequently, there is a pressing need to develop taxonomic frameworks that standardize terminology and provide a solid scientific foundation for future studies.

A clear knowledge gap also exists between Arab and international research. European scholarship has advanced considerably in developing diverse practical tools such as digital assessments and audiovisual recordings, whereas Arab research remains largely confined to traditional methods, such as surveys, often focused on conventional media.

International studies further demonstrate the varied impact of digital media literacy across different audience groups, particularly in enhancing their critical and analytical skills, and in expanding the integration of artificial intelligence applications into literacy education.

By contrast, Arab studies, while emphasizing the importance and value of media literacy, indicate that it has yet to be effectively implemented within educational and media institutions. This disconnection renders many applied research outcomes impractical and ineffective. Moreover, previous studies show that media literacy plays a vital role in reinforcing trust in institutions, supporting civic engagement, and promoting adherence to public measures during crises such as pandemics. However, the persistent inability of users to verify information shared on social media remains a significant challenge. This underscores the need for more comprehensive initiatives that address local contextual differences and aim to bridge the digital divide.

### 3.2 Theoretical framework

#### 3.2.1 Digital media literacy

Digital Media Literacy (DML) represents an evolution and extension of traditional media literacy, emerging as one of the key pillars of modern education due to the extensive integration of digital media into everyday life and learning environments (Al Adwan et al., 2025; EL Semary, 2025). It is defined as “the ability to access, analyze, evaluate, create, and engage with digital content in a critical and ethical manner” (Abdelhay et al., 2025; Dezuanni, 2016).

Another definition describes it as “an individual's conscious ability to interact with digitally produced content in its various forms and to participate in its creation through skills of access, comprehension, analysis, evaluation, and responsible participation” (Ahmed, 2025).

UNESCO (2013) defines digital media literacy as a set of essential competencies that enable individuals to access, understand, use, create, and share information through digital means in order to foster effective participation in civic and democratic life.

Digital media literacy integrates three overlapping domains:

- Media Literacy
- Information Literacy
- Digital Literacy

Additionally, media literacy has been defined as “the ability to identify media content sources, their political, social, commercial, and cultural purposes, and the context in which they are produced and received; it encompasses the critical analysis, interpretation, and production of media materials and the understanding of the values embedded within them” (Abdelghani, 2022).

The diversity of definitions presented in the literature reflects the conceptual fluidity of digital media literacy and highlights a central theoretical challenge within the field (Dadakhonov, 2024). While these definitions converge on core competencies such as access, analysis, creation, and critical evaluation, they often remain disconnected from broader behavioral, cognitive, or sociotechnical theories. This conceptual fragmentation is not merely definitional but signals a deeper issue related to the limited theoretical anchoring of digital media literacy research.

In the broader field of media, communication, and technology studies, several well-established theoretical models, such as the Technology Acceptance Model (TAM), the Unified Theory of Acceptance and Use of Technology (UTAUT), Uses and Gratifications Theory (UGT), Media Dependency Theory (MDT), Social Cognitive Theory (SCT), Protection Motivation Theory (PMT), and Critical Media Literacy (CML) frameworks have been extensively employed to explain media use, technological adoption, risk perception, learning processes, and critical engagement with media systems (Ashi, 2017; Al-Zou'bi, 2025; Trejo-Quintana et al., 2025; Fayez et al., 2026).

However, the literature reviewed indicates that these models are rarely employed explicitly within digital media literacy research. When referenced, they are often mentioned in passing or detached from the empirical design, resulting in a predominantly descriptive body of research that prioritizes skill measurement over theoretical explanation.

#### 3.2.2 Historical development of the concept

The concept of media literacy has evolved from its traditional form into what is now known as digital media literacy, in response to the shifting nature of media consumption and production environments (Lu et al., 2024). This evolution can be traced through three main phases, as illustrated in Table 1.

**Table 1:** Evolution of the concept of media literacy.

**Period**	**Core features**
1970s−1980s	Focus on analyzing traditional media messages (television, newspapers) and developing critical awareness.
1990s–mid-2000s	Integration of technology into education and an emphasis on information literacy.
2010–present	Focus on digital interaction, digital citizenship, fact-checking, and developing skills for media production and participation.

The historical evolution outlined in Table 1 demonstrates that the development of media literacy has largely followed technological change rather than theoretical innovation. While the shift toward digital participation and verification reflects new media realities, it has not been accompanied by a corresponding development of integrative theoretical frameworks capable of explaining users' cognitive, social, and behavioral engagement with digital media.

#### 3.2.3 Components of digital media literacy

The literature indicates that digital media literacy includes several key components (Farahani et al., 2024; Moon and Bai, 2020), including:

Although the components summarized in Table 2 provide a comprehensive skills-based model of digital media literacy, they remain largely operational rather than explanatory. The emphasis on competencies such as access, verification, and engagement frequently substitutes for theoretical grounding, thereby reinforcing an instrumental understanding of digital media literacy at the expense of deeper epistemic and sociocultural analysis.

**Table 2:** Core components of digital media literacy.

**Component**	**Concept**
Access	The ability of individuals to use digital media tools.
Critical thinking	Understanding and interpreting messages within their cultural and political context.
Creation	Creating digital content (textual, visual, or audio) using digital tools.
Engagement	Engaging consciously and responsibly in digital discussions.
Verification	The ability to distinguish between accurate and misleading information.
Digital safety	Awareness of privacy issues and online security risks.

## 4 Methodology

### 4.1 Approach

The present study adopts a critical, journal-based scoping analysis rather than a second-order or a systematic review in the strict meta-analytical sense. While the concept of second-order analysis is acknowledged as a valuable meta-research approach, this study does not claim to fulfill all of its methodological requirements. Instead, it employs an interpretive and exploratory qualitative design aimed at selected research trends, theoretical orientations, and methodological patterns within selected, thematically specialized publication contexts.

The analytical logic of the study is grounded in qualitative synthesis through categorical comparison, focusing on how digital media literacy research is constructed, framed, and operationalized within specific academic journals. Accordingly, the study prioritizes epistemic interpretation and trend identification over statistical generalization or exhaustive field coverage.

### 4.2 Research method

This study adopts a qualitative secondary data analysis approach, drawing on the principles of critical scoping analysis rather than systematic review protocols such as PRISMA. The selection of journals was purposive, aiming to enable an in-depth qualitative comparison between Arab and international publication contexts rather than comprehensive field coverage. Consequently, the findings are analytical and illustrative rather than statistically generalized.

The study does not follow PRISMA guidelines, as it is not a systematic review designed for comprehensive field coverage. Instead, it adopts a scoping-oriented qualitative synthesis, which allows for greater analytical flexibility and critical interpretation of theoretical and methodological trends.

The study adopts a qualitative approach appropriate to the humanities and social sciences, which examines the details of accumulated scholarship to produce a general description of the phenomenon under study. This approach is judged to be most suitable for formulating scientific directions that underpin future research. The method used is A critical scoping analysis, which permits in-depth qualitative analysis of the scholarly corpus and focuses on tracing the significance of changes in the phenomenon over a specified period in theoretical, methodological, and procedural terms. It also enables careful mapping of dominant research currents and the extent to which they contribute to cumulative knowledge and to theoretical and methodological development within media studies.

### 4.3 Population and sample

The study population comprises all peer-reviewed articles published in academic journals (the study sample) that address the topic of “digital media literacy.” The research examined scholarly output on digital media literacy published over a 10-year period (2016–mid-2025) in four peer-reviewed journals specializing in media studies. The sample included two leading Arab journals focusing on media research, both indexed in regional and international databases such as the Egyptian Knowledge Bank (EKB), DOAJ, the Arab Citation Index (Arcif), and ERIC. It also included two international journals specializing in media literacy, indexed in Scopus. This ensured a high level of scientific rigor and academic peer review for the analyzed studies.

For this study, “international” journals are defined as those published in English and indexed internationally; they are not intended to represent all global or European research traditions. The four journals selected for the sample were:

1. Media Research Journal (Egypt): Published quarterly by the Faculty of Mass Communication at Al-Azhar University, this journal is one of the earliest Arab journals dedicated to media studies, with its first issue published in October 1993. It covers diverse research in media and communication.
2. The Egyptian Journal of Media Research: Published quarterly by the Faculty of Mass Communication at Al-Azhar University, this journal is one of the earliest Arab journals dedicated to media studies, with its first issue published in October 1993. It covers diverse research in media and communication.
3. Journal of Media Literacy Education (USA): Published by the National Association for Media Literacy Education, this journal provides a forum for interdisciplinary research on both established topics (e.g., media and children, critical pedagogy, educational policy) and emerging areas (e.g., data literacy, digital mobility, multiliteracies). The journal has been indexed in Scopus since 2019.
4. International Journal of Media and Information Literacy (USA): Published by Cherkas Global University Press, this journal features empirical and theoretical work on media and information literacy, including media culture, media competence, and media education. The journal has been indexed in Scopus since 2016.

To provide a clearer understanding of the publication volume and the potential scale of article screening, the publication frequency of the selected journals was identified. Media Research Journal and The Egyptian Journal of Media Research are published on a quarterly basis (four issues per year). The Journal of Media Literacy Education publishes three issues annually, while the International Journal of Media and Information Literacy publishes two issues per year. This variation in publication frequency was taken into account during the manual screening process across the study period.

Regarding language scope, the two Arab journals primarily publish articles in Arabic, with English titles and abstracts provided for international accessibility. The two international journals publish exclusively in English. Only full-text articles written in Arabic or English were considered eligible for inclusion in the analysis.

This study employed a purposive sampling approach to select four peer-reviewed academic journals for a comparative analysis of two publishing environments: Arab journals specializing in media research and international journals specializing in media literacy. This approach is based on the methodological assumption that specialized journals constitute stable knowledge spaces that reflect research priorities, peer-review standards, and prevailing theoretical trends, enabling in-depth qualitative analysis beyond superficial quantitative description. The exclusion of high-impact international journals or interdisciplinary periodicals was not a dismissal of their scientific value, but a methodological decision aligned with the analytical and critical aim of this study. Including large databases such as Web of Science would have shifted the study toward a comprehensive, systematic, or bibliometric review, which differs fundamentally from the current exploratory and critical approach focusing on in-depth reading within specific publication contexts.

Selection criteria for the sample included:

- Peer-review status and a focus on media studies with a regular publication schedule.
- Explicit or implicit attention to topics related to digital media literacy.
- Publication in either Arabic or English.

Explicit relevance was defined as studies that directly addressed digital media literacy or closely related concepts in their titles, keywords, or stated objectives. Implicit relevance referred to studies that examined competencies or practices conceptually aligned with digital media literacy without using the term explicitly.

The screening process relied on a set of core keywords, including: (digital media literacy, media literacy, information literacy, social media literacy, digital citizenship, critical evaluation of online content, misinformation detection, and fact-checking skills).

All issues of the four selected journals published between 2016 and mid-2025 were manually browsed to identify potentially relevant studies. The initial screening yielded a total of 974 published articles across the four journals during the study period. Titles and abstracts were first reviewed to assess relevance to digital media literacy. Articles that met the preliminary relevance criteria were then examined in full text. After applying the inclusion and exclusion criteria, a final sample of 78 articles was retained for analysis. The study relied on secondary data analysis through a comprehensive survey of all peer-reviewed studies on digital media literacy published in the selected journals during the specified timeframe. Studies were manually collected according to inclusion criteria, ensuring direct relevance to digital media literacy, peer-review status, and publication within the study timeframe.

The collected studies were categorized and analyzed using a content analysis form designed specifically for this research, incorporating rigorous subject, methodological, and theoretical variables. Percentages were calculated by dividing the number of studies in each analytical category by the total number of studies in each journal and by the total number of studies (78 studies) to ensure clarity and accuracy in presenting the results.

### 4.4 Data collection instruments

The study relied primarily on literature analysis as its primary research tool. This approach enabled a comprehensive, in-depth analytical examination of the phenomenon under investigation, rather than merely providing a historical overview.

For this purpose, the study employed a directed content analysis tool designed specifically for analyzing previous studies. This instrument was developed based on methodological literature on the evaluation of scholarly output and subsequently validated by a panel of professors in media studies.

The analysis framework comprised the following dimensions:

- Publication information (year, country, journal, institution)
- Topical focus of the study (educational, media, cultural, etc.)
- Disciplinary domain (philosophical, psychological, sociological, etc.)
- Theoretical frameworks employed
- Research design or methodological approach (quantitative, qualitative, mixed-methods, etc.)
- Geographical scope of the study (Europe, Asia, the Americas, the Arab world, etc.)
- Sampling type (probability, non-probability)
- Type of media platform analyzed (traditional media, digital journalism, social networks, etc.)
- Data collection tools (questionnaire, interview, content analysis, document analysis, multiple tools)
- Research setting (educational, community-based, etc.)
- Target population (children, youth, general audience, etc.)
- Key findings and recommendations

### 4.5 Scope of the study

• Thematic Scope: The study was limited to research on digital media literacy that was published in peer-reviewed journals and available in full text.

• Time Scope: The analysis covered the period from 2016 to mid-2025. Data for the year 2025 represent partial-year publications available at the time of analysis. Accordingly, trends observed this year should be interpreted cautiously and not taken as indicative of full-year patterns.

• Language Scope: The study included only research published in Arabic and English.

## 5 Results

This section of the study presents the key findings from the content analysis of studies on digital media literacy collected between 2016 and mid-2025 in the following academic journals:

- Media Research Journal: 12 studies were analyzed.
- Egyptian Journal of Media Research: 14 studies were analyzed.
- Journal of Media Literacy Education: 22 studies were analyzed.
- International Journal of Media and Information Literacy: 30 studies were analyzed.

**Figure 1:**
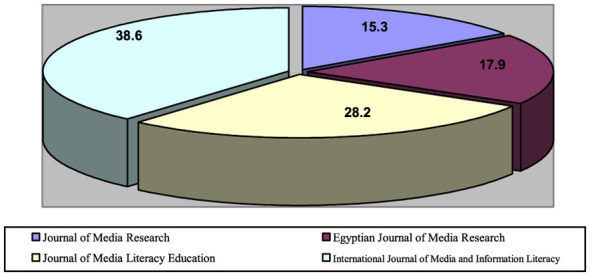
Percentage of studies by journal.

As shown in Figure 1, a total of 78 studies were analyzed across the four selected journals. The majority were published in international journals. The *International Journal of Media and Information Literacy* accounted for the largest share of studies, with 30 studies (38.6% of the total), followed by the *Journal of Media Literacy Education*, with 22 studies (28.2%). In contrast, the number of studies in Arab journals was lower: the *Egyptian Journal of Media Research* included 14 studies (17.9%), and the *Journal of Media Research* published 12 studies (15.3%). This distribution highlights the greater interest of international journals in media literacy research, reflecting the growing importance of media literacy amid ongoing transformations in communication and digital media.

Table 3 shows that 2024 had the highest number of studies on media literacy among the years studied, accounting for 20.5% of the total sample, followed by 2025 (19.2%) and 2022 (14.1%), respectively. This trend indicates a noticeable increase in research interest in media literacy during the early 2020s.

**Table 3:** Year of publication of the analyzed studies in the selected journals.

**Year of publication**	**Journal of media research**		**Egyptian journal of media research**		**Journal of media literacy education**		**International journal of media and information literacy**		**Total**	
	**Frequency**	**%**	**Frequency**	**%**	**Frequency**	**%**	**Frequency**	**%**	**Frequency**	**%**
2016	1	8.3	0	0.0	2	9.1	4	13.3	7	9.0
2017	1	8.3	0	0.0	2	9.1	1	3.3	4	5.1
2018	0	0.0	0	0.0	0	0.0	2	6.7	2	2.6
2019	1	8.3	0	0.0	0	0.0	1	3.3	2	2.6
2020	1	8.3	2	14.3	0	0.0	5	16.7	8	10.3
2021	3	25.0	1	7.1	0	0.0	3	10.0	7	9.0
2022	2	16.7	4	28.6	3	13.6	2	6.7	11	14.1
2023	1	8.3	4	28.6	1	4.5	0	0.0	6	7.7
2024	0	0.0	2	14.3	7	31.8	7	23.3	16	20.5
Mid-2025	2	16.7	1	7.1	7	31.8	5	16.7	15	19.2

As shown in Table 4, most studies in the selected journals fall within the educational field (76.9%), followed by the media (67.9%) and the cultural fields (35.9%). This distribution underscores the strong association between media literacy research and the educational domain, particularly within universities, which serve as the primary institutions promoting media literacy. The research field of each article was determined based on a review of the title, keywords, and abstract. When necessary, the full text was examined to ensure accurate classification according to the primary focus of the study.

**Table 4:** Research fields of the analyzed studies in the selected journals.

**Research field “topic”**	**Journal of media research**		**Egyptian journal of media research**		**Journal of media literacy education**		**International journal of media and information literacy**		**Total**	
	**Frequency**	**%**	**Frequency**	**%**	**Frequency**	**%**	**Frequency**	**%**	**Frequency**	**%**
Educational	9	75.0	4	28.6	21	95.5	26	86.7	60	76.9
Media	9	75.0	0	0.0	16	72.7	28	93.3	53	67.9
Cultural	0	0.0	1	7.1	11	50.0	16	53.3	28	35.9
Psychological	0	0.0	0	0.0	1	4.5	1	4.5	2	2.6
Sports	0	0.0	0	0.0	0	0.0	0	0.0	0	0.0
Health	1	8.3	0	0.0	3	13.6	2	6.7	6	7.7
Social	2	16.7	2	14.3	1	4.5	1	3.3	6	7.7
Technological	0	0.0	2	14.3	6	27.3	8	26.7	16	20.5
Interdisciplinary	0	0.0	7	50.0	0	0.0	0	0.0	7	9.0

**Figure 2:**
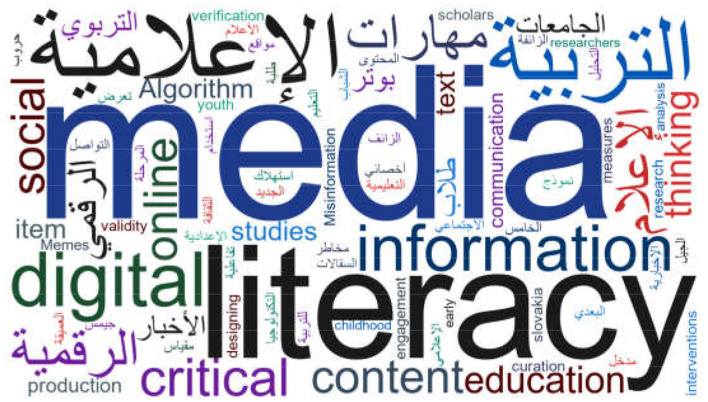
Word cloud of keywords associated with the thematic trends of the analyzed research.

Figure 2 shows the word cloud keywords associated with the thematic trends of the analyzed research:

The concentration of research topics within the educational and skill-based dimensions of digital media literacy suggests a functional understanding of the concept, often detached from broader socio-political, cultural, or critical perspectives. This thematic narrowing may reflect the dominance of policy-driven and curriculum-oriented agendas, especially in contexts where media literacy is framed primarily as a protective or corrective mechanism rather than a critical social practice.

According to Table 5, the theoretical orientation ranked first (59%), followed by the technological orientation (56.4%), and finally, the psychological orientation (9%). This distribution reflects the interdisciplinary nature of media literacy, which integrates theoretical, technical, and psychological dimensions.

**Table 5:** Research orientations of the analyzed studies.

**Research orientation**	**Journal of media research**		**Egyptian journal of media research**		**Journal of media literacy education**		**International journal of media and information literacy**		**Total**	
	**Frequency**	**%**	**Frequency**	**%**	**Frequency**	**%**	**Frequency**	**%**	**Frequency**	**%**
Theoretical	2	16.7	6	42.9	12	54.5	26	86.7	46	59.0
Psychological	0	0.0	1	7.1	4	18.2	2	6.7	7	9.0
Technological (AI, etc.)	10	83.3	7	50.0	12	54.5	15	50.0	44	56.4

Theoretical orientation was identified based on the explicit presence of a theoretical framework referenced in the literature review or conceptual section of each article. Studies that did not explicitly state or apply a theoretical model were classified as having “no explicit theoretical framework.” The classification focused on the primary theoretical lens guiding the study's design or interpretation.

Table 6 indicates that approximately 73.1% of the reviewed studies did not explicitly articulate a formal theoretical framework guiding their research design or analytical interpretation. This finding does not imply a complete absence of theoretical influence; rather, it suggests a prevailing reliance on descriptive or concept-driven approaches in which theory is often implicit, underdeveloped, or treated as a background reference rather than as an analytical engine, while 7.7% of the studies adopted the Media Literacy Theory, followed by Symbolic Interactionism (3.8%), and Media Dependency Theory, and Technology Acceptance Theory (2.6%) each. This may be attributed to the fact that some researchers perceive media literacy as a theoretical entry point rather than a fully developed conceptual framework.

**Table 6:** Theories adopted by the analyzed studies.

**Theory**	**Journal of media research**		**Egyptian journal of media research**		**Journal of media literacy education**		**International journal of media and information literacy**		**Total**	
	**Frequency**	**%**	**Frequency**	**%**	**Frequency**	**%**	**Frequency**	**%**	**Frequency**	**%**
Media literacy theory	0	0.0	5	35.7	0	0.0	1	3.3	6	7.7
Media dependency theory	1	8.3	1	7.1	0	0.0	0	0.0	2	2.6
Technology acceptance theory	0	0.0	1	7.1	1	4.5	0	0.0	2	2.6
Uses and gratifications theory	0	0.0	0	0.0	0	0.0	1	3.3	1	1.3
Technological determinism	0	0.0	1	7.1	0	0.0	0	0.0	1	1.3
Instructional scaffolding	1	8.3	0	0.0	0	0.0	0	0.0	1	1.3
Symbolic interactionism	1	8.3	1	7.1	0	0.0	1	3.3	3	3.8
Protection motivation theory	1	8.3	0	0.0	0	0.0	0	0.0	1	1.3
Theory of action	0	0.0	0	0.0	1	4.5	0	0.0	1	1.3
Problem-solving theory	0	0.0	0	0.0	0	0.0	1	3.3	1	1.3
Parental mediation theory	0	0.0	1	7.1	0	0.0	0	0.0	1	1.3
Social responsibility theory	0	0.0	1	7.1	0	0.0	0	0.0	1	1.3
No theory	6	50.0	4	28.6	20	90.9	27	90.0	57	73.1

For the purposes of this analysis, a “theoretical framework” is operationally defined as the explicit and systematic use of a recognized theory or conceptual model that guides the formulation of research questions, the interpretation of findings, or the explanation of media-related phenomena. General conceptual references, definitional discussions, or implicit theoretical assumptions were not classified as constituting a formal theoretical framework.

As shown in Table 7, the most commonly used research approach was quantitative (53.8%), followed by qualitative (25.6%) and mixed-methods (20.6%). This indicates a continuing preference for quantitative designs in both Arab and international research traditions, while qualitative and mixed approaches remain less frequently employed.

**Table 7:** Research methodologies followed by the analyzed studies.

**Research methodology**	**Journal of media research**		**Egyptian journal of media research**		**Journal of media literacy education**		**International journal of media and information literacy**		**Total**	
	**Frequency**	**%**	**Frequency**	**%**	**Frequency**	**%**	**Frequency**	**%**	**Frequency**	**%**
Quantitative	11	91.7	9	64.3	9	40.9	13	43.3	42	53.8
Qualitative	0	0.0	1	7.1	8	36.4	11	36.7	20	25.6
Mixed-methods	1	8.3	4	28.6	5	22.7	6	20.0	16	20.6

Table 8 shows that the research tools in the analyzed studies were mainly questionnaires (41%), followed by multiple tools (23.1%) and document-based approaches (20.5%). This distribution reflects the predominance of field studies, in which questionnaires are the most suitable data-collection method.

**Table 8:** Research tools used in the analyzed studies.

**Research tools**	**Journal of media research**		**Egyptian journal of media research**		**Journal of media literacy education**		**International journal of media and information literacy**		**Total**	
	**Frequency**	**%**	**Frequency**	**%**	**Frequency**	**%**	**Frequency**	**%**	**Frequency**	**%**
Questionnaire	7	58.3	8	57.1	6	27.3	11	36.7	32	41.0
Quantitative content analysis	1	8.3	0	0.0	0	0.0	0	0.0	1	1.3
Qualitative analysis guide	0	0.0	0	0.0	1	4.5	0	0.0	1	1.3
Interview	2	16.7	1	7.1	2	9.1	1	3.3	6	7.7
Scale	0	0.0	0	0.0	4	18.2	0	0.0	4	5.1
Documents/reports	0	0.0	0	0.0	5	22.7	11	36.7	16	20.5
Multiple tools	2	16.7	5	35.7	4	18.2	7	23.3	18	23.1

Table 9 indicates that non-Arab studies accounted for about 65.4% of the sample. Arab studies ranked first (34.6%), followed by European studies (26.9%) and Asian studies (20.5%). This reflects the high number of publications from the two American journals included in the sample. Geographical scope was determined based on the study setting or the location of the research sample as reported in the methodology section. When the study context was not explicitly specified, the geographical focus was inferred from the institutional or national context described by the authors.

**Table 9:** Geographical scope of the analyzed studies.

**Geographical scope of research**	**Journal of media research**		**Egyptian journal of media research**		**Journal of media literacy education**		**International journal of media and information literacy**		**Total**	
	**Frequency**	**%**	**Frequency**	**%**	**Frequency**	**%**	**Frequency**	**%**	**Frequency**	**%**
Arab studies	12	100.0	12	85.7	2	9.1	1	3.3	27	34.6
European studies	0	0.0	1	7.1	9	40.9	11	36.7	21	26.9
African studies	0	0.0	1	7.1	0	0.0	0	0.0	1	1.3
Asian studies	0	0.0	0	0.0	4	18.2	12	40.0	16	20.5
American studies	0	0.0	0	0.0	7	31.8	6	20.0	13	16.7

Table 10 shows that non-probability sampling was the most frequently used (73.1%), compared to probability sampling (26.9%). This suggests that most of the analyzed studies aim not to generalize results to broader populations but rather to offer indicative insights and exploratory findings that reflect the growing academic interest in media literacy as an emerging scientific approach, especially amid rapid communication and digital transformations.

**Table 10:** Types of research samples used in the analyzed studies.

**Research sample type**	**Journal of media research**		**Egyptian journal of media research**		**Journal of media literacy education**		**International journal of media and information literacy**		**Total**	
	**Frequency**	**%**	**Frequency**	**%**	**Frequency**	**%**	**Frequency**	**%**	**Frequency**	**%**
Probability sample	3	25.0	8	57.1	5	22.7	5	16.7	21	26.9
Non-probability sample	9	75.0	6	42.9	17	77.3	25	83.3	57	73.1

Table 11 shows that digital media ranked first (92.3%), followed by traditional media (83.3%). This finding demonstrates a clear shift in media literacy research toward digital media education, reflecting the field's evolution in response to technological and communicative transformations.

**Table 11:** Types of media used in the analyzed studies.

**Type of media**	**Journal of media research**		**Egyptian journal of media research**		**Journal of media literacy education**		**International journal of media and information literacy**		**Total**	
	**Frequency**	**%**	**Frequency**	**%**	**Frequency**	**%**	**Frequency**	**%**	**Frequency**	**%**
Digital media	10	83.3	14	100.0	20	90.9	28	93.3	72	92.3
Traditional media	10	83.3	13	92.9	19	86.4	23	76.7	65	83.3

Table 12 indicates that most studies were conducted in educational settings (61.5%), followed by those targeting the general public (26.9%) and virtual environments (11.5%). This finding aligns with the results shown in Table 5, which demonstrated that the educational domain ranked first among the main focus areas of media literacy research.

**Table 12:** Research population in the analyzed studies.

**Research population**	**Journal of media research**		**Egyptian journal of media research**		**Journal of media literacy education**		**International journal of media and information literacy**		**Total**	
	**Frequency**	**%**	**Frequency**	**%**	**Frequency**	**%**	**Frequency**	**%**	**Frequency**	**%**
Education (school, university, etc.)	10	83.3	12	85.7	13	59.1	13	43.3	48	61.5
General public	2	16.7	1	7.1	5	22.7	13	43.3	21	26.9
Virtual environment	0	0.0	1	7.1	4	18.2	4	13.3	9	11.5

As shown in Table 13, the most frequently targeted group in the analyzed studies was youth (50%), followed by the general public (30.8%), adolescents (10.3%), and children (9%). This corresponds with the findings in Table 13, which revealed that educational contexts were the predominant focus of research on media literacy. The category “Youth” refers to adolescents and young adults, including university students, unless a study explicitly specified a different age classification.

**Table 13:** Target groups in the analyzed studies.

**Target group**	**Journal of media research**		**Egyptian journal of media research**		**Journal of media literacy education**		**International journal of media and information literacy**		**Total**	
	**Frequency**	**%**	**Frequency**	**%**	**Frequency**	**%**	**Frequency**	**%**	**Frequency**	**%**
Children	1	8.3	0	0.0	4	18.2	2	6.7	7	9.0
Adolescents	0	0.0	0	0.0	4	18.2	4	13.3	8	10.3
Youth	11	91.7	11	78.6	9	40.9	8	26.7	39	50.0
General public	0	0.0	3	21.4	5	22.7	16	53.3	24	30.8

**Figure 3:**
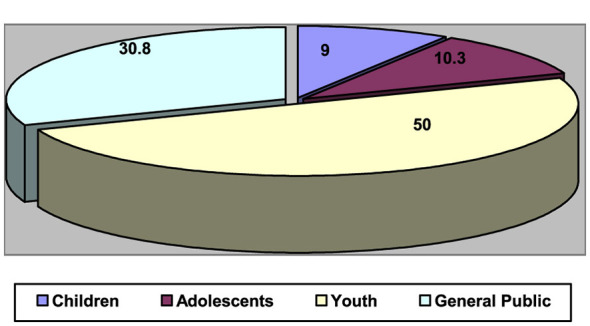
Target group in the studies analyzed.

Although the study relies on specific journals, the analysis goes beyond simply describing editorial policies or enumerating scholarly output. It extends to analyzing the methodological and theoretical frameworks governing research in digital media literacy, such as the nature of research questions, methodological design patterns, and the extent to which theoretical frameworks are employed. Thus, the study is not merely a periodic review of journals, but a critical analysis of knowledge-production trends within the field. Figure 3 illustrates the overall percentages in the Table 13.

## 6 Discussion

Rather than treating the tabulated results as isolated statistical indicators, the discussion section interprets these patterns as manifestations of deeper epistemological, institutional, and contextual dynamics shaping digital media literacy research across different publication environments.

While international journals demonstrate greater methodological diversity and theoretical articulation, this difference should not be interpreted as a linear indicator of scholarly “advancement.” Rather, it reflects structural disparities in research funding, academic training, publication expectations, and evaluation regimes. Arabic research, although more descriptive, often addresses context-specific educational and cultural challenges that are underrepresented in international scholarship.

### 6.1 Discussion of findings from studies published in Arabic journals

The findings of studies and research published in the Arabic journals included in the sample indicate that media literacy has become a strategic approach to addressing the challenges of the digital environment and the associated risks posed by fourth- and fifth-generation information wars. Several studies emphasized the need to integrate specialized university programs and courses in media literacy (Ashi, 2017; Sayed Ali, 2020) and to develop national strategies for embedding this field within curricula at various educational levels (Mohamed, 2016). The need to establish specialized centers for media literacy and to coordinate efforts among relevant institutions was also highlighted to ensure complementarity of roles (Abdel Aty, 2021).

The studies further stressed the importance of equipping students with skills in producing, editing, and publishing digital content across multiple platforms, while enhancing their ability to verify information and their awareness of the cultural risks posed by modern media wars (Al-Suraity, 2025; ghander, 2023; Al-Ghaly, 2022). Findings from some studies revealed that students' levels of conscious and critical thinking remain moderate and that entertainment content continues to be the most preferred among digital platform users, underscoring the need to raise levels of digital media literacy (Al-Suraity, 2025; korany, 2020).

Moreover, the studies showed that parents play a crucial role in promoting media awareness by monitoring the content their children are exposed to Ramadan (2023). The main motivation for parents' participation in media awareness programs was their desire to control the type of content directed at their children (Abdelghani, 2022; Al-Rabbabeh, 2022).

In comparative terms, the findings demonstrated that international journals are more advanced in the field of digital media literacy than their Arabic counterparts. They have developed more innovative practical tools such as digital interviews, cognitive tests, and audiovisual recordings (Ahmad Tajuddin et al., 2024). These studies also covered a broader age range, including older adults, and placed strong emphasis on collaborative research and innovation in educational program design (EL Semary, 2025; Bin Lagh and Nassar, 2023).

Statistically, some studies found a significant correlation between media literacy and the ability to combat misinformation, though no clear relationship was found between media literacy and variables such as field of study or income level. It was also found that media literacy fosters civic engagement; however, universities and news websites still play a limited role in promoting media culture, as reflected in students' modest possession of advanced digital skills (Mekawey et al., 2021; Mohamed, 2021). Several studies further noted that traditional media curricula were incompatible with distance education during the COVID-19 pandemic, particularly practical courses and graduation projects. This highlights the need to redesign such curricula to align with the requirements of the digital environment (Hanafi, 2023).

Overall, the topics addressed in digital media literacy studies largely reflect the media and educational landscape in the Arab world, particularly in light of the rapid digital transformation Arab societies have witnessed over the past decade. The focus of numerous studies on issues such as media literacy in education, critical engagement with social media platforms, and fact-checking skills aligns with the real challenges facing Arab educational and media institutions in the era of the open digital space.

For example, the growing research interest in artificial intelligence and digital technologies, such as combating fake news and disinformation, clearly reflects the realities of the Arab media environment. In recent years, this environment has seen a widespread proliferation of unreliable content, especially during political and health crises. This problem became particularly evident during the COVID-19 pandemic, as numerous media reports and field studies revealed a weakness in digital verification skills among large segments of the Arab public. This explains the emphasis in academic studies on developing critical awareness and evaluating information sources (Babacan et al., 2025; Salman and Fayyad, 2025; Fayez Abdelhay, 2021).

The abundance of studies addressing the role of digital media literacy in schools and universities aligns with the educational policies of several Arab countries, which have begun integrating digital citizenship concepts and new media skills into their curricula and educational activities. This is evident in educational initiatives aimed at enhancing students' proficiency in using digital media safely and responsibly, which explains the aptness of choosing this topic as a key focus in the studies analyzed. Furthermore, the topics related to Arab youth's use of social media platforms such as Facebook, Twitter, and Instagram reflect a tangible social reality: the increasing reliance on these platforms as primary sources of news and public interaction. This phenomenon has prompted numerous studies to focus on digital media literacy as a tool to mitigate potential negative impacts, such as polarization, hate speech, and the uncritical influence of circulating content.

Conversely, the limited number of studies addressing the profound political and economic dimensions of digital media literacy reveals a gap between the complexity of the Arab reality and the prevailing research agenda. Despite the growing role of digital media in shaping political awareness and social mobilization in the Arab region, these issues remain underrepresented in the literature. This underscores the urgent need to redirect future research toward topics more closely linked to power structures, cultural contexts, and social transformations in the Arab world. Based on the above, it can be argued that the topics addressed in the analyzed studies largely reflect real priorities within the Arab context. However, they also reveal an imbalance in research approaches, where the educational and skills-based dimensions often overshadow broader critical and societal perspectives. This finding underscores the importance of expanding the scope of digital media literacy studies in the future to enhance their capacity to interpret and engage with the complex Arab reality more comprehensively and accurately.

### 6.2 Discussion of findings from studies published in international journals

The findings of the reviewed studies indicate that the field of media and information literacy encompasses a wide range of overlapping concepts, such as media literacy, information literacy, and digital literacy (Wuyckens et al., 2022). However, this conceptual diversity is marked by a lack of consensus regarding precise definitions and the specific competencies each of these literacies entails. This ambiguity has led to theoretical confusion and hindered systematic knowledge accumulation (Yelubayeva and Gabdullina, 2024). Such discrepancies are largely attributed to the varying disciplinary and theoretical backgrounds of researchers, as well as the absence of comprehensive overviews of these perspectives in the literature.

The findings further highlighted the need for integrative conceptual frameworks or analytical taxonomies to help organize these intersecting notions and facilitate the development of accurate and reliable measurement tools. Experimental instruments such as media literacy tests and self-perception measures (SPMIL) have demonstrated high levels of validity and reliability (Ahmad Tajuddin et al., 2024; Çela et al., 2025; Demir and Akar, 2024), supporting their usefulness in assessing the media and information literacy skills of students and the general public across different contexts.

It was also found that levels of media and information literacy vary according to social, cultural, age-related, and educational factors (Davidson et al., 2025). Some studies reported that students demonstrated advanced levels of digital competence and readiness to apply these skills in public life (Leal-Rico, 2024), whereas others noted limited public awareness of critical thinking and fact-checking tools, particularly in underprivileged or rural communities (Botturi et al., 2025). Recurrent findings also noted significant intergenerational gaps in media and digital competencies, with a noticeable decline in these skills with age (Alexander and Galina, 2020).

Another notable trend was the growing emphasis on integrating new dimensions such as *digital well-being* into media literacy education, thereby broadening its goals to include promoting psychological health and digital safety alongside cognitive and communicative development (Feerrar, 2022). Some studies even revealed a positive correlation between excessive smartphone use and higher levels of media literacy (Okela, 2022), suggesting opportunities to redirect digital engagement toward educational and constructive purposes.

Moreover, findings showed that embedding media and information literacy in educational curricula enhances digital production and creativity skills—such as video creation—and supports active and experiential learning methods (Rojas-Estrada and Sánchez-Vilela, 2024; Good and Ciccone, 2025). Nonetheless, challenges persist regarding teacher preparation and the absence of effective assessment strategies. Digital learning experiences, such as MOOCs, were found to contribute to the spread of media literacy (Tropiano and Reis, 2024), though these initiatives remain difficult to categorize and require more adaptive strategies for diverse learner groups.

From a socio-political standpoint, results revealed that media literacy is closely tied to public trust in government, support for public policy, and compliance with health measures during pandemics—demonstrating its essential role in shaping public awareness and promoting civic participation (Vu, 2024). Conversely, other studies found low levels of media literacy among audiences, particularly in fact-checking on social networks (Marwiyah et al., 2025), highlighting the need for more inclusive, context-sensitive media and information literacy initiatives.

The relative distribution of topics in digital media literacy studies is not merely a matter of abstract quantitative indicators; it directly reflects research priorities linked to real-world transformations in educational, media, and societal environments. The high percentage of studies focusing on the educational and media dimensions of digital media literacy, which constituted the largest proportion of the total studies analyzed, can be explained by the increasing challenges facing educational systems in dealing with contemporary digital environments and the demands they impose regarding the development of critical thinking skills, information verification, and conscious engagement with media content.

For example, the abundance of studies addressing the integration of digital media literacy into educational curricula reflects a direct response to the widespread use of social media platforms among students and the resulting real-world issues such as misinformation, hate speech, and exposure to unreliable content. These studies demonstrate a practical interest in preparing learners to analyze media messages more effectively and understand the political and cultural contexts in which they are produced, aligned with the requirements of digital citizenship in contemporary societies.

Conversely, the low percentage of studies addressing the political, economic, or cultural dimensions of digital media literacy points to a clear research gap between the complex social reality in which digital media operate and the prevailing academic interest. Despite the increasing influence of digital media in shaping political awareness, directing public opinion, and reproducing patterns of symbolic dominance, these aspects remain underrepresented in the literature under analysis, particularly in the Arab context.

Furthermore, the limited number of studies addressing digital media literacy from a societal or critical perspective largely reflects the nature of prevailing educational and research policies, which tend to focus on short-term, practical, skills-based aspects at the expense of broader critical approaches that link media literacy to social, economic, and authoritarian structures. This is evident in the scarcity of studies addressing issues such as media power, ideology, or digital inequality, despite their strong presence in the contemporary digital landscape.

Therefore, the disparity in the percentages of these topics not only reflects researchers' preferences but also points to institutional and methodological trends governing knowledge production in the field of digital media literacy. Based on these findings, there is a need to expand the research agenda in the future so that it goes beyond a narrow educational focus, to include more real-world issues related to the social, political, and cultural context in which digital media operate, thereby enhancing the critical role of media literacy in building a more inclusive societal awareness.

Overall, these findings underscore the necessity for a more integrated approach to media and information literacy, one that acknowledges conceptual plurality, supports the development of comprehensive measurement tools, and embeds these skills within educational and community practices. They also stress the importance of fostering collaboration between academic, political, and social institutions to narrow the digital divide and enhance citizens' capacities to navigate the growing cognitive and communicative challenges of the digital age.

## 7 Conclusions

This study aimed to provide an in-depth analytical overview of recent trends in digital media literacy research by examining published literature, particularly studies published between 2016 and mid- 2025, in selected Arab and international journals. The analysis focused on the theoretical and methodological frameworks, thematic orientations, and target groups represented in the field. Based on this comprehensive analysis, the main findings can be summarized as follows:

- The results show that media literacy has become an urgent necessity requiring strategic integration among educational, media, and community institutions. This can be achieved by embedding media literacy across all levels of education, designing specialized university courses, and establishing dedicated centers to promote media literacy and coordinate efforts among relevant stakeholders. Emphasis should also be placed on equipping students with skills for producing, editing, and sharing digital content, alongside the development of their critical and analytical abilities to counter misinformation and digital propaganda, two of the most pressing challenges in today's media landscape.
- Many studies found that students still exhibit only moderate levels of spontaneous critical awareness and that entertainment content remains the most attractive to them, posing a significant challenge to efforts aimed at fostering critical engagement and discernment.
- A clear gap was observed between Arab and English research traditions. American studies, in particular, were found to be more advanced in developing practical tools—such as digital tests and audiovisual recordings—and in addressing diverse age groups, including older adults. By contrast, Arab studies tend to remain focused on more traditional frameworks. Statistically, some studies revealed a positive relationship between media literacy and the ability to counter rumors, while no significant correlation was found with academic specialization or income level.
- The findings further confirm the role of media literacy in promoting civic participation. However, limited involvement of universities and news platforms in promoting media literacy has led to students' inadequate mastery of digital communication skills (Bano et al., 2024). Moreover, no significant differences were found between males and females or between students at public and private universities in acquiring media literacy skills (Khalifa et al., 2026), suggesting that these skills have become essential for all individuals in the modern digital society. Traditional media curricula were also found to be poorly suited for remote learning during the COVID-19 pandemic, particularly courses involving practical components such as graduation projects—underscoring the need to redesign these curricula to align with digital learning requirements.
- Arab studies emphasized the importance and value of media literacy and its societal impact. However, this emphasis remains largely theoretical, with limited implementation in educational and media institutions, which diminishes the real-world effectiveness of these findings.
- English studies, on the other hand, revealed greater diversity regarding the effects of digital media literacy. While some found significant improvements in audiences' analytical and critical skills, others argued that genuine digital media literacy remains underdeveloped amid the rapid spread of digital technologies, the expansion of disinformation, and the increasing sophistication of AI-driven manipulation. These studies concluded that the challenges facing digital media literacy are growing more complex.
- The results indicate that the conceptual multiplicity surrounding media, information, and digital literacy remains a major obstacle to building cumulative knowledge and unified theoretical models. The absence of consensus among researchers regarding key definitions and competencies contributes to confusion in setting educational and social goals. This aligns with Mohamed (2024), who observed that conceptual fluidity in this field can obscure the purpose of educational outcomes. Hence, there is a pressing need to develop taxonomic frameworks that harmonize terminology and provide a sound theoretical foundation for future studies.
- Experimental assessment tools, such as SPMIL and media literacy tests, have demonstrated high validity and reliability, confirming their potential usefulness across educational contexts. However, these tools must be culturally and contextually adapted, since variations in sociocultural environments can influence how effectively they capture audiences' actual skills.
- The findings reveal clear variations in media literacy levels across age groups, educational backgrounds, and social environments. These variations are consistent with Vu (2024), who noted generational gaps and a decline in digital proficiency with age. This highlights the need for educational programs tailored to cultural and demographic diversity. Furthermore, limited critical thinking skills in certain contexts call for a stronger integration of fact-checking and evaluative analysis within educational and media curricula.
- In the field of education, findings affirm that integrating media literacy into curricula enhances digital production and creativity skills. Nevertheless, the lack of adequate teacher training and assessment strategies limits its impact. This aligns with (Rojas-Estrada and Sánchez-Vilela 2024), who emphasized that the success of media literacy depends on teachers' ability to apply it in practice, not just in theory.
- On the social and political level, results show that media literacy strengthens public trust in institutions, promotes civic participation, and supports adherence to public measures during crises such as pandemics. However, audiences' persistent difficulty verifying information on social media remains a significant challenge, underscoring the need for broader, context-sensitive initiatives to address the digital divide.
- In conclusion, these findings underscore the need for an integrated approach that combines theoretical and practical dimensions of media literacy. Such an approach should prioritize the development of culturally relevant measurement tools, flexible educational models, and interdisciplinary collaboration. This would help cultivate a critically aware public capable of confronting the growing cognitive and communicative challenges of the digital era. Current research on digital media literacy remains in its early stages and still lacks sufficient empirical tools to assess its real impact, highlighting the importance of continued context-driven inquiry in this vital field.

## 8 Recommendations

Based on the findings of this study, the following recommendations are proposed:

1. Strengthening the core competencies of media literacy and promoting the effective use of both traditional and new media to exercise democratic rights, fulfill civic responsibilities, and develop the ability to access, analyze, evaluate, create, and innovate.
2. Conducting in-depth studies on integrating media literacy into social networks and new media platforms. Media literacy is essential for everyone and requires new strategies, skills, and clearly defined roles and responsibilities.
3. Fostering continuous dialogue among stakeholders concerned with media literacy through social networks. Media literacy policy calls for independent, high-quality research that is subject to ongoing and systematic evaluation.
4. Investing in the new communicative environment provided by social networks for the benefit of individuals and society by adopting innovative strategies that help utilize these platforms to develop media literacy skills.
5. Integrating media literacy skills into all levels of education to cultivate critical thinking abilities across educational stages and enable effective engagement with modern communication tools, new media, and social networks.

## 9 Limitations and future research

### 9.1 Limitations

This study is subject to several limitations that should be considered when interpreting its findings:

- First, the analysis is based on a purposive selection of four peer-reviewed journals rather than a comprehensive database-driven sampling. While this design enables in-depth qualitative comparison between Arabic and English-language publication contexts, it limits the generalizability of the results beyond these specific scholarly environments.
- Second, the study's findings are to be understood within their analytical and qualitative framework. They do not aim to make statistical generalizations about the field of digital media literacy as a whole, but rather to provide analytical indicators that help in understanding research trends within specific publishing contexts. These indicators can serve as a comparative framework for broader studies in the future.
- Third, the review does not follow formal systematic review protocols such as PRISMA. This reflects a deliberate methodological choice aligned with critical scoping analysis; however, it constrains the study's capacity to claim exhaustive coverage of the literature.
- Fourth, the identification of theoretical frameworks was limited to explicitly stated models. Studies relying on implicit or loosely articulated theoretical assumptions may therefore be underrepresented in the theoretical classification.
- Finally, the temporal scope includes partial data for 2025, and findings related to this year should be interpreted with caution.

### 9.2 Future research directions

- Future research should expand sampling strategies to include large-scale academic databases (Web of Science) to support field-level synthesis and enhance representativeness. Integrating qualitative scoping approaches with bibliometric and network-based methods (using VOSviewer or Publish or Perish) may further strengthen analytical rigor.
- There is also a clear need for studies that move beyond descriptive mapping toward explicit theory-building and theory-testing in digital media literacy research, particularly within Arab contexts. Longitudinal and cross-cultural comparative designs would contribute to a more cumulative and conceptually coherent body of scholarship in this evolving field.

(*) List of Experts Who Reviewed the Research Instrument:

- Prof. Ahmed Samir Hammad—Professor of Journalism, Al-Azhar University, Egypt.
- Prof. Hatem Ali Al-Salhi—Professor of Mass Communication, Sana'a University, Yemen.
- Prof. Adel Fahmy—Professor of Mass Communication, Faculty of Mass Communication, Cairo University, Egypt.
- Prof. Abed Al-Rab Al-Nabi—Professor of Journalism, Imam Muhammad Ibn Saud University, Saudi Arabia.
- Prof. Abdulrahman Al-Nami—Professor of Public Relations, Imam Muhammad Ibn Saud University, Saudi Arabia.
- Prof. Mohamed Hossam El-Din—Professor of International Communication, Faculty of Mass Communication, Cairo University, Egypt.
- Prof. Mohamed Saad Ibrahim—Professor of Mass Communication, Minia University, Egypt.

## Data Availability

The original contributions presented in the study are included in the article/supplementary material, further inquiries can be directed to the corresponding author.
